# Gut bacteriobiota and mycobiota are both associated with Day-28 mortality among critically ill patients

**DOI:** 10.1186/s13054-022-03980-8

**Published:** 2022-04-13

**Authors:** Renaud Prevel, Raphaël Enaud, Arthur Orieux, Adrian Camino, Patrick Berger, Alexandre Boyer, Laurence Delhaes, Didier Gruson

**Affiliations:** 1 Medical Intensive Care Unit, CHU Bordeaux, 33000 Bordeaux, France; 2 Centre de Recherche Cardio-Thoracique de Bordeaux, Inserm UMR 1045, Univ Bordeaux, 33000 Bordeaux, France; 3 CRCM Pédiatrique, CIC 1401, CHU Bordeaux, 33000 Bordeaux, France; 4 Mycology-Parasitology Department, CIC 1401, CHU Bordeaux, 33000 Bordeaux, France

**Keywords:** Microbiota, Mycobiota, Intensive care unit

## Abstract

**Introduction:**

Gut microbiota is associated with host characteristics such as age, sex, immune condition or frailty and is thought to be a key player in numerous human diseases. Nevertheless, its association with outcome in critically ill patients has been poorly investigated. The aim of this study is to assess the association between gut microbiota composition and Day-28 mortality in critically ill patients.

**Methods:**

Rectal swab at admission of every patient admitted to intensive care unit (ICU) between October and November 2019 was frozen at − 80 °C. DNA extraction was performed thanks to QIAamp^®^ PowerFecal^®^ Pro DNA kit (QIAgen^®^). V3–V4 regions of 16SRNA and ITS2 coding genes were amplified by PCR. Sequencing (2x250 bp paired-end) was performed on MiSeq sequencer (Illumina^®^). DADA2 pipeline on R software was used for bioinformatics analyses. Risk factors for Day-28 mortality were investigated by logistic regression.

**Results:**

Fifty-seven patients were consecutively admitted to ICU of whom 13/57 (23%) deceased and 44/57 (77%) survived. Bacteriobiota *α*-diversity was lower among non-survivors than survivors (Shannon and Simpson index respectively, *p* < 0.001 and *p* = 0.001) as was mycobiota *α*-diversity (respectively *p* = 0.03 and *p* = 0.03). Both gut bacteriobiota and mycobiota Shannon index were independently associated with Day-28 mortality in multivariate analysis (respectively OR: 0.19, 97.5 CI [0.04–0.60], *p* < 0.01 and OR: 0.29, 97.5 CI [0.09–0.75], *p* = 0.02). Bacteriobiota *β*-diversity was significantly different between survivors and non-survivors (*p* = 0.05) but not mycobiota *β*-diversity (*p* = 0.57). Non-survivors had a higher abundance of *Staphylococcus haemolyticus*, *Clostridiales* sp., *Campylobacter ureolyticus*, *Akkermansia* sp., *Malassezia sympodialis*, *Malassezia dermatis* and *Saccharomyces cerevisiae*, whereas survivors had a higher abundance of *Collinsella aerofaciens*, *Blautia* sp., *Streptococcus* sp., *Faecalibacterium prausnitzii* and *Bifidobacterium* sp.

**Conclusion:**

The gut bacteriobiota and mycobiota *α* diversities are independently associated with Day-28 mortality in critically ill patients. The causal nature of this interference and, if so, the underlying mechanisms should be further investigated to assess if gut microbiota modulation could be a future therapeutic approach.

**Supplementary Information:**

The online version contains supplementary material available at 10.1186/s13054-022-03980-8.

## Introduction

The emergence of next-generation sequencing during the last decade has allowed the exploration of the gut microbiota role in human health and diseases. The gut microbiota is composed of microbes from different kingdoms (bacteria, fungus, archae, virus). The number of microbes composing this microbiota is approximately equivalent to the number of our own human cells [1]. Critical illness is known to have profound effects on gut microbiota with extreme dysbiosis of gut microbiota identified in critically ill patients [2, 3]. Despite a large interpersonal variation in gut microbiota dysregulation, critically illness is consistently associated with decreased diversity of Actinobacteria, decreased abundance of butyrate-producing bacteria and commensal *Firmicutes* and *Bacteroidetes*, while the abundance of opportunistic pathogens is increased [4]. Host impact on gut microbiota during critical illness is mediated by both endogenous and external factors [5]. Endogenous factors include increased production of opioids and catecholamines, decreased bile-salt concentration, gastrointestinal dysmotility and loss of epithelial integrity in the intestine. External factors include antibiotics, proton pump inhibitor, enteral/parenteral feeding, sedatives, opioids and catecholamines. Extreme dysbiosis of gut microbiota during critical illness is thought to have numerous and profound effects on host metabolism including decreased systemic short-chain fatty acid levels, impaired immune condition and to increase the risk of super-infection, of acute kidney injury (AKI) or of muscle wasting [5]. Despite those data derived from in vitro and animal studies and trials focused on non-critical human diseases, only one study assessed the association between gut microbiota and death in critically ill patients. It suggested that bacteriobiota *α*-diversity and the presence of *Enterococcaceae* family could be associated with death in critically ill patients [6]. The aim of this study was thus to assess the association between gut microbiota composition and Day-28 mortality in critically ill patients.

## Patients and methods

### Patients inclusion and data collection

Every consecutive patient older than 18 years of age admitted to the medical intensive care unit (ICU) at Bordeaux University Hospital in October and November 2019 was prospectively screened to participate to Microbe study (NCT04131569) and included in this ancillary analysis.

Data were prospectively recorded by physicians in charge of the patient by questioning the patients, patients’ family and patients’ general practitioners. Electronic worksheet was completed by two medical intensive care residents. Comorbidities were defined as follows: chronic obstructive pulmonary disease and asthma were defined according to lung function testing. Chronic heart failure was defined according to transthoracic echocardiography and chronic coronary disease based on stress test or percutaneous coronary intervention. Other comorbidities included history of chronic kidney disease (glomerular filtration rate < 60 mL/min/1.73 m^2^), immunosuppression (drugs, haematological disease, blood marrow transplantation, solid organ transplantation, plasma exchanges indicated by autoimmune disorders, human immunodeficiency virus infection) and the worse simplified acute physiology score II (SAPSII) within the first 24 h following admission. Acute respiratory distress syndrome was defined according to Berlin’s criteria [7], septic shock according to Sepsis-3 definition [8] and AKI to KDIGO guidelines [9].

### Samples collection and preparation for microbiota analysis

The rectal swab (Transport Swab VWR, Copan^®^) performed for faecal ESBL-E carriage screening at admission before administration of antimicrobial agents was collected and frozen at − 80 °C. DNA extraction was performed by QIAamp^®^ PowerFaecal^®^ Pro DNA kit (QIAgen^®^). A step of mechanical lysis (2 cycles of 30 s at 7000 rpm on Precellys evolution) was added just after the chemical lysis of the kit. V3–V4 regions of 16SRNA coding gene and ITS2 were amplified by PCR as previously reported [10]. Sequencing (2x250 bp paired-end) was performed on MiSeq sequencer (Illumina^®^) at the Bordeaux Transciptome Genome platform (INRAe, France).

### Bioinformatics analysis

DADA2 pipeline on R software was used for bioinformatics analyses [11]. DADA2 pipeline was preferred as it allows inter-studies comparison (if identical primers are used for amplification) [11] and is more accurate for mycobiota analysis [12]. We defined bacteriobiota as the bacterial kingdom of the microbiota and mycobiota as the fungal kingdom of the microbiota. Gut bacteriobiota and mycobiota *α*-diversity was expressed by Shannon index, Simpson index and evenness. Between sample beta-diversity differences (measured using Bray Curtis dissimilarity) were tested using a permutational multivariate ANOVA (Permanova) from “vegan” package with 10,000 permutations, while accounting for individual identity as a covariate. Gut bacteriobiota and mycobiota *α-* and *β*-diversities were compared thanks to “Phyloseq” package on R software v3.6.0. Linear discriminant analysis (LDA) effect size (LefSe) analysis was performed from microbiomeMarker package. We used mock communities to avoid a non-efficient sequencing experiment, and negative controls to identify and remove potential reagent contaminants of bacterial and fungal microbiota with the microDecon R package [13]. Comparison of *β*-diversity between negative control, mock community and samples is available in Additional file 1: Figs. S1 and S2 for bacteriobiota and mycobiota respectively). The final average read counts were 66,434 (standard deviation ± 17,634) for 1285 bacterial ASVs and 3647 (standard deviation ± 1267) for 361 fungal ASVs. The 16S rRNA gene and ITS2 sequences have been submitted to the European Nucleotide Archive (Accession N◦ ERP134948).

### Statistical analysis

No statistical sample size calculation was performed a priori, and sample size was equal to the number of patients admitted to ICU with available rectal swab during the study period. Quantitative variables are presented as median and interquartile range (IQR) and compared by use of the Mann–Whitney Wilcoxon rank-sum test. Categorical variables are expressed as number of patients (percentage) and compared by use of the Chi-square or Fisher’s test. Risk factors for Day-28 mortality were investigated by logistic regression. First, a univariate analysis was performed. Only variables with a *p* value < 0.10 were included in the multivariate analysis. Variables comprised in SAPSII such as malignancy, sepsis (blood pressure), ARDS (Pa02/FiO2 ratio) and acute kidney injury (urine output, serum urea level and kaliemia) were not included in the analysis as it is already known that they are associated with SAPSII.

All statistical tests were 2-tailed, and statistical significance was defined as *p* < 0.05. Statistical analyses were assessed by the R 3.6.0 statistical software (R foundation for Statistical Computing Vienna, Austria).

### Ethics

According to French law and the French Data Protection Authority, the handling of these data for research purposes was declared to the Data Protection Officer of the Bordeaux University Hospital. The study obtained the approval of the Institutional Review Board of the Bordeaux University Hospital (declaration number CER BDX-2021-36). Patients (or their relatives, if any) were notified about the anonymized use of their healthcare data via the department's booklet.

## Results

### Flow chart and patients’ characteristics

**Fig. 1:**
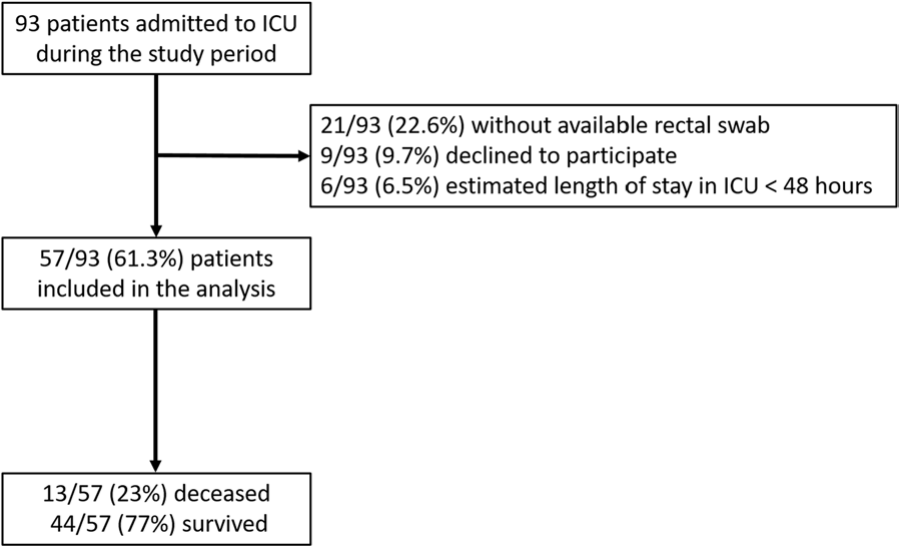
Flow chart. *ICU* intensive care unit

Ninety-three patients were prospectively screened during the study period. Twenty-one of them (22.6%) did not have any swab available either due to lack of screening or swab without any stool on it, 9 declined to participate (9.7%), and 6 (6.5%) had an estimated length of stay of less than 48 h (Fig. 1). Among the 57 patients with rectal swabs available for microbiota analysis, 13 (23%) deceased within the first 28 days following admission to ICU and 44 (77%) survived. Patients’ characteristics are summarized in Table 1. Non-survivors were more often male (12/13 (92.9%) vs. 27/44 (61.4%), *p* = 0.04) with active solid cancer (4/13 (28.6%) vs. 2/44 (4.5%), *p* = 0.02) and higher SAPSII score (78 [75–87] vs. 61 [46–74], *p* < 0.01).

**Table 1:** Patients’ characteristics and comparison between survivors and non-survivors

	Total (*n* = 57)	Non-survivors (*n* = 13)	Survivors (*n* = 44)	*p* value
Characteristics at admission to ICU				
Age	75 [65–79]	73 [65–79]	76 [64–79]	0.99
Sex (male)	39 (68.4%)	12 (92.9%)	27 (61.4%)	0.04
SAPSII	68 [49–78]	78 [75–87]	61 [46–74]	< 0.01
Septic shock	20 (35.1%)	7 (50%)	13 (29.5%)	0.18
ARDS	8 (14%)	4 (28.6%)	4 (9.1%)	0.07
Acute kidney injury	32 (56.1%)	12 (85.7%)	20 (45.5%)	< 0.01
Comorbidities				
Chronic respiratory disease	24 (42.1%)	5 (35.7%)	19 (43.2%)	0.52
COPD	13 (22.8%)	3 (21.4%)	10 (22.7%)	1.00
Asthma	6 (10.5%)	1 (7.1%)	5 (11.4%)	1.00
Chronic heart failure	28 (49.1%)	9 (64.3%)	19 (43.2%)	0.12
Chronic coronary disease	22 (38.6%)	7 (50%)	15 (34.1%)	0.22
Chronic kidney disease	13 (22.8%)	4 (28.7%)	9 (20.5%)	0.47
Immunosuppression	12 (21%)	3 (21.4%)	9 (20.5%)	1.00
Active solid cancer	6 (10.5%)	4 (28.6%)	2 (4.5%)	0.02
Proton pump inhibitor	15 (26.3%)	3 (21.4%)	12 (27.3%)	1.00
Metformin	6 (10.5%)	4 (28.6%)	6 (13.6%)	0.21
Antimicrobial treatment during the 3 previous months	19 (33.3%)	3 (21.4%)	16 (36.4%)	0.51
Treatment				
Mechanical ventilation	32 (56.1%)	10 (71.4%)	22 (50%)	0.12
Renal replacement therapy	10 (17.5%)	6 (42.9%)	4 (9.1%)	< 0.01

Results are presented as proportion for categorical variables and median [interquartile range] for continuous variables

*p* values are for comparison between survivors and non-survivors. Threshold for statistical significance: *p* = 0.05

*ARDS* acute respiratory distress syndrome, *ICU* intensive care unit, *SAPS* simplified acute physiology score II

### Gut bacteriobiota and mycobiota α-diversities are significantly decreased in non-survivors compared with survivors

**Fig. 2:**
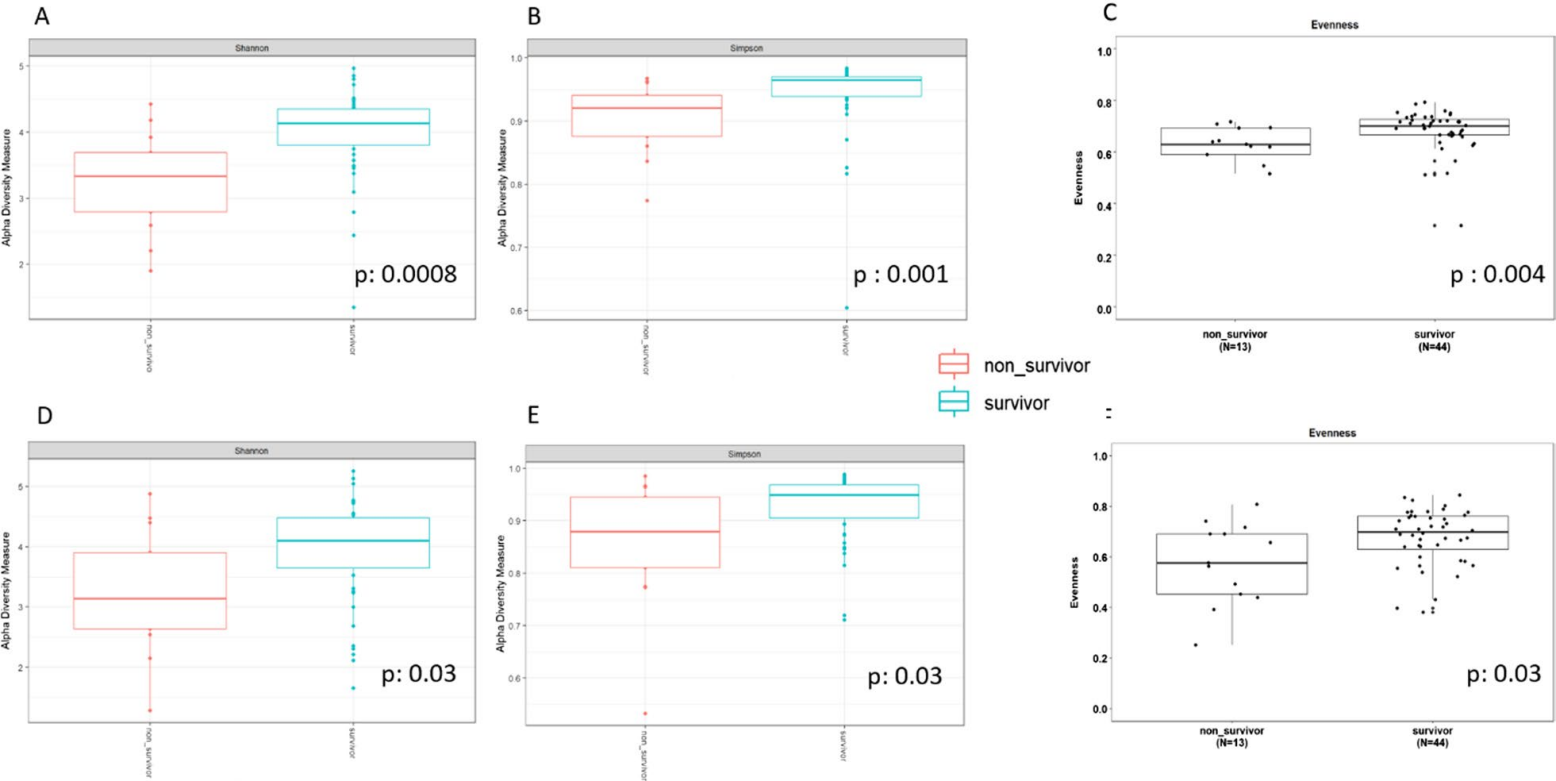
Comparison of gut microbiota *α-*diversities between survivors (in blue) and non-survivors (in red). Gut bacteriobiota *α-*diversity according to Shannon index (**A**), Simpson index (**B**) and evenness (**C**). Gut mycobiota *α* diversity according to Shannon index (**D**), Simpson index (**E**) and evenness (**F**). Threshold for statistical significance: *p* = 0.05

Non-survivors had lower gut bacteriobiota *α-*diversity (expressed by Shannon or Simpson index and evenness respectively, *p* < 0.001, *p* = 0.001 and *p* < 0.01) (Fig. 2A, B, C) than survivors as well as well as lower gut mycobiota *α-*diversity (expressed by Shannon or Simpson index and evenness, *p* = 0.03 for all those 3 index) (Fig. 2D, E, F).

### Both gut bacteriobiota and mycobiota α-diversities are independently associated with Day-28 mortality in critically ill patients

After univariate analysis, gut bacteriobiota Shannon index, gut mycobiota Shannon index, sex, SAPSII, history of atrial fibrillation, current malignancy, sepsis at admission to ICU, ARDS at admission to ICU and acute kidney injury at admission to ICU were associated with Day-28 mortality (Table 2). Current malignancy, sepsis at admission to ICU, ARDS at admission to ICU and acute kidney injury at admission to ICU were not included in the multivariate analysis as they are included in SAPSII items.

**Table 2:** Factors associated with Day-28 mortality in critically ill patients

Variables	Univariate analysis OR	97.5 CI	*p* value
**Shannon 16S rDNA**	**0.26**	**[0.09–0.62]**	**< 0.01**
**Shannon ITS2**	**0.47**	**[0.23–0.91]**	**0.03**
Age	1.02	[0.97–1.08]	0.50
**Sex (male)**	**7.56**	**[1.3–144]**	**0.06**
**SAPSII**	**1.07**	**[1.02–1.14]**	**0.01**
Chronic pulmonary disease	0.82	[0.22–2.87]	0.76
COPD	1.02	[0.20–4.15]	0.98
Chronic heart disease	2.96	[0.83–12.3]	0.11
**Atrial fibrillation**	**3.33**	**[0.88–12.7]**	**0.07**
Coronary disease	2.26	[0.64–8.21]	0.20
Chronic kidney disease	1.73	[0.40–6.76]	0.44
**Malignancy**	**13.1**	**[2.39–104]**	**< 0.01**
Immunosuppression	1.17	[0.23–4.83]	0.84
Long-term proton pump inhibitor	0.89	[0.18–3.59]	0.87
Long-term metformin	1.01	[0.98–1.03]	0.99
Antibiotics within the past 3 months	0.53	[0.11–2.07]	0.39
Septic shock at admission	2.78	[0.78–10.3]	0.11
**Acute respiratory distress syndrome at admission**	**4.44**	**[0.90–22.4]**	**0.06**
**Acute kidney injury at admission**	**14.4**	**[2.51–274]**	**0.01**
Digestive infection within the past 3 months	1.75	[0.08–19.8]	0.66

Variables	Multivariate analysis OR	97.5 CI	*p* value
**Shannon 16S rDNA**	**0.19**	**[0.04–0.60]**	**< 0.01**
**SAPSII**	**1.08**	**[1.01–1.17]**	**0.04**
**Sex (male)**	**14.4**	**[1.45–516]**	**0.05**
Atrial fibrillation	5.03	[0.76–41.1]	0.10

Variables	Multivariate analysis OR	97.5CI	*p* value
**Shannon ITS2**	**0.29**	**[0.09–0.75]**	**0.02**
**SAPSII**	**1.08**	**[1.02–1.17]**	**0.02**
**Sex (male)**	**18.8**	**[1.84–582]**	**0.04**
Atrial fibrillation	4.53	[0.07–33.4]	0.11

Bold in univariate analysis: variables assessed for inclusion in the multivariate analysis. Bold in multivariate analysis: variables independently associated with Day-28 mortality

*ICU* intensive care unit, *ITS2* internal transcribed spacer 2, *OR* odds ratio, *SAPSII* simplified acute physiology score II, *16S rDNA* DNA region coding for ribosomal 16S RNA subunit, *97.5 CI* 97.5% confidence interval

After multivariate analysis, both gut bacteriobiota and mycobiota Shannon index remain independently associated with Day-28 mortality (respectively OR: 0.19, 97.5 CI [0.04–0.60], *p* < 0.01 and OR: 0.29, 97.5 CI [0.09–0.75], *p* = 0.02) (Table 2).

### Non-survivors and survivors have significantly dissimilar bacteriobiota but not mycobiota

**Fig. 3:**
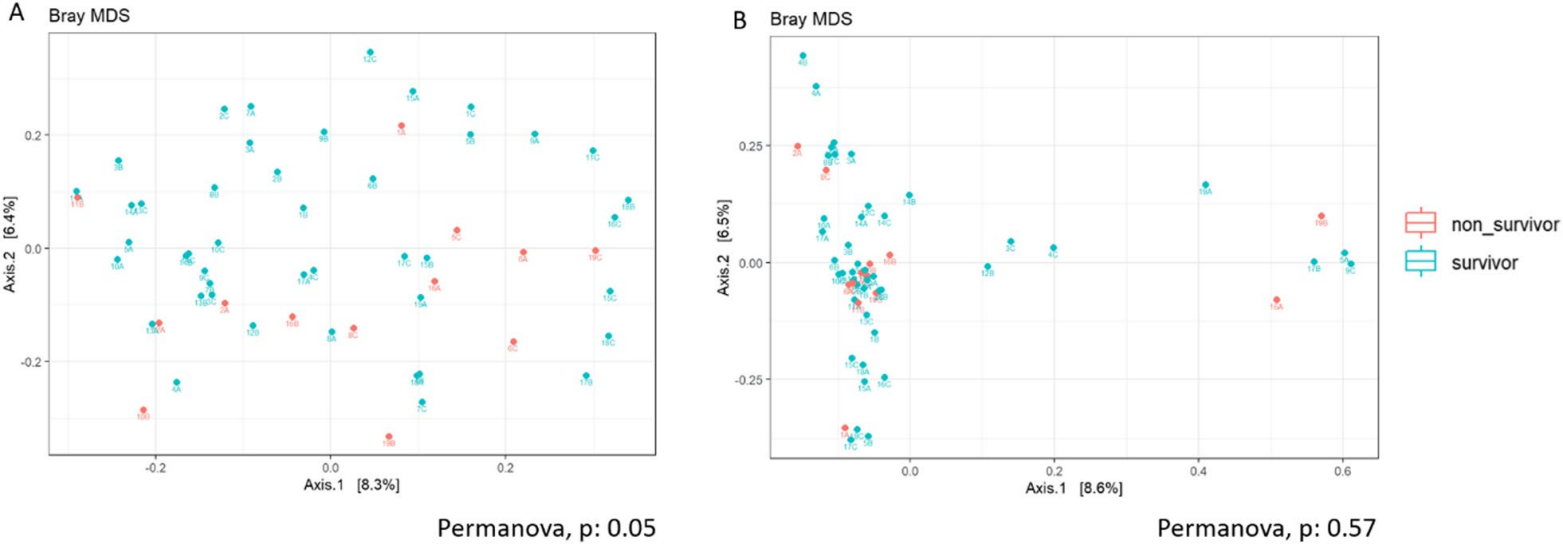
Comparison of gut microbiota similarity (*β-*diversities) between survivors and non-survivors. Metric Bray–Curtis analysis of *β*-diversity for gut bacteriobiota (**A**) and gut mycobiota (**B**). Red: non-survivors. Green: survivors. Threshold for statistical significance: *p* = 0.05

Gut bacteriobiota *β*-diversity was significantly different between survivors and non-survivors in ICU (Permanova, *p* = 0.05) but not mycobiota *β*-diversity (Permanova, *p* = 0.57) (Fig. 3A, B).

### Identification of bacterial and fungal species associated with survival

**Fig. 4:**
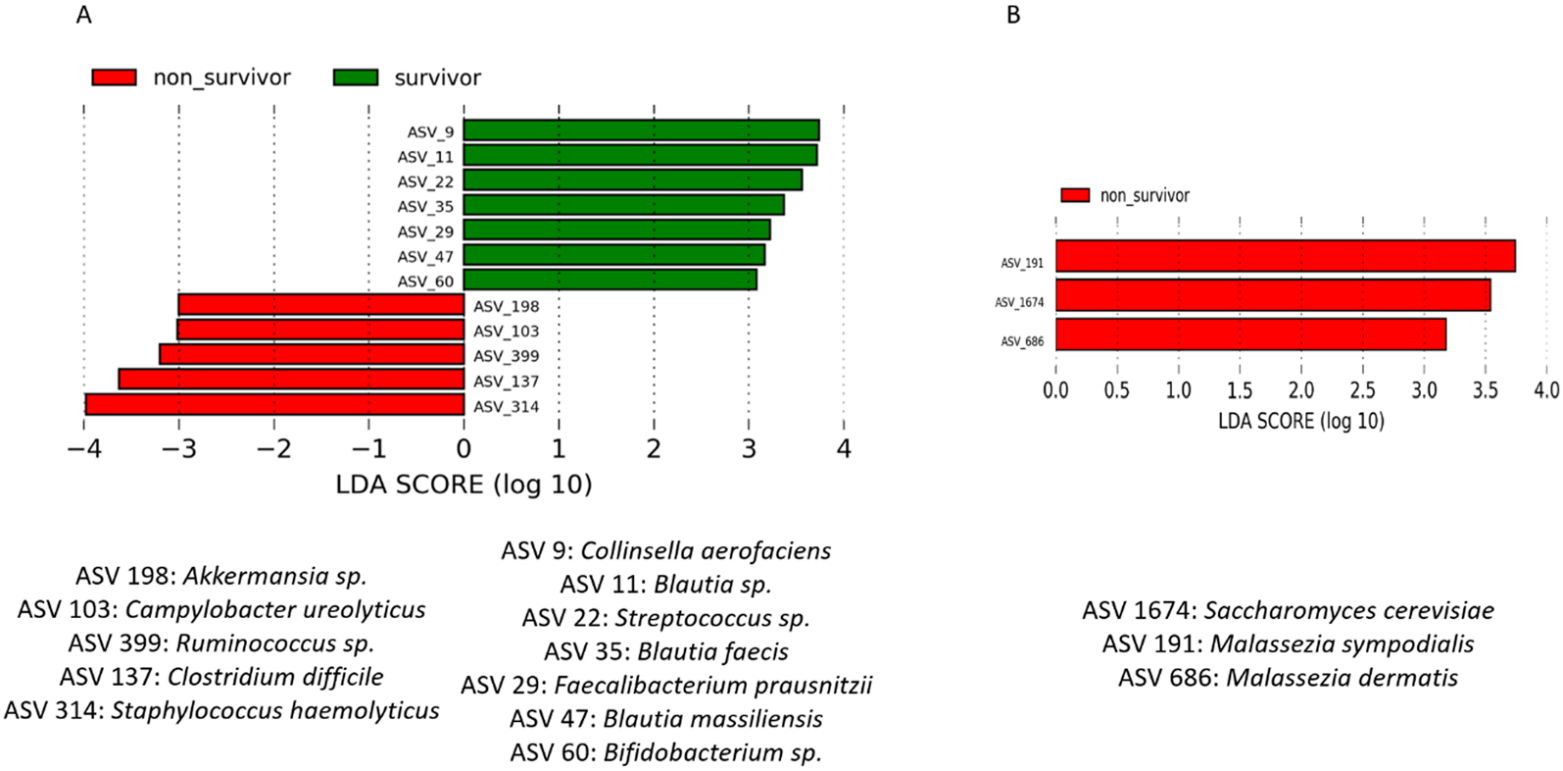
Microbial species associated with mortality. LefSe analysis with linear discriminant analysis (LDA) for bacterial species (**A**) and fungal species (**B**). Threshold for statistical significance: LDA > 3log. *ASV* amplicon sequence variant

Non-survivors had a higher abundance (LDA > 3log) in gut microbiota of *Staphylococcus haemolyticus*, *Clostridiales* sp., *Campylobacter ureolyticus*, *Akkermansia* sp., *Malassezia sympodialis*, *Malassezia dermatis* and *Saccharomyces cerevisiae* whereas survivors had a higher abundance of *Blautia* sp., *Streptococcus* sp., *Faecalibacterium prausnitzii* and *Bifidobacterium* sp. (Fig. 4A, B).

## Discussion

In critically ill patients, gut bacteriobiota and mycobiota *α* diversities are decreased in non-survivors compared to survivors. Gut bacteriobiota was dissimilar (*β-*diversity) between non-survivors and survivors but not mycobiota. Moreover, our study is the first to assess the association of both gut bacteriobiota and mycobiota *α*-diversities with fatal outcome in ICU patients and both are independently associated with Day-28 mortality. This result is not surprising as commensal fungi have been demonstrated to recapitulate the protective benefits of intestinal bacteria. In fact, mannans, a highly conserved component class of fungal cell walls, stimulate local and systemic immunity and protect mice depleted of commensal bacteria from colitis and influenza A virus infection [14]. Fungal kingdom of microbiota should not be under-investigated in future studies.

Few studies investigated the impact of gut microbiota on ICU patients’ outcome and mostly focused on identifying biomarkers instead of analysing *α* diversity. In addition, they focused on the bacterial kingdom (bacteriobiota) exclusively. A first cohort study of 98 neurocritically ill patients indicates that the gut bacteriobiota composition differs significantly from that in a healthy population. In 50 neurocritically ill patients of this cohort, the magnitude of dysbiosis increased during the first week in the neurological ICU with an increase in gut *Enterobacteriales* burden which was associated with a 92% increased risk of mortality at Day 180 [15]. Similarly, gut bacteriobiota in sepsis and septic shock patients had an increased abundance of microbes tightly associated with inflammation, such as *Parabacteroides*, *Fusobacterium* and *Bilophila* species and evidenced a remarkable loss of microbial diversity during the ICU stay. The increase in abundance of pathogenic species, such as *Enterococcus spp.,* was differentially increased in sepsis patients who died [16]. Other biomarkers derived from gut microbiota could be the abundance of *Bifidobacterium* in gut bacteriobiota [17], a higher abundance being associated with survival, or the progression of imbalance in the ratio of *Bacteroidetes* to *Firmicutes* within the first 7 days [18].

Three studies investigated the role of lung microbiota in ICU patients, still focusing on bacteriobiota. A first study with 29 endotracheal aspirates demonstrated a negative correlation between lung bacteriobiota *α-*diversity and APACHE II score [19]. A second study demonstrated that lung bacterial burden, but not bacteriobiota *α-*diversity, was associated with ventilator-free days in 91 critically ill patients receiving mechanical ventilation [20]. The last study included 36 mechanically ventilated patients with extra-pulmonary sepsis (thus excluding patients with lung infections) and suggested that lung bacterial *α-*diversity could predict ICU mortality [21].

A major limitation is to know whether there is any causal inference in this association between gut and lung microbiota and clinical outcomes in critically ill patients, but several data suggest that there could be a causal relationship. In fact, gut microbiota dysbiosis is known to have various deleterious effects on the host. Locally, it could increase microbial virulence and favour microbial translocation in systemic and lymphatic circulation [5]. Antibiotic-induced changes in the gut microbiota have been demonstrated to be associated with decreased neutrophils maturation in the bone marrow, decreased splenic B1 B lymphocytes production and IgM production, decreased dendritic cells migration to the lungs and other numerous systemic immunity impairment [22]. Thus, the absence of some protective microbial species or the expansion of some deleterious microbial species in gut microbiota associated with ICU stay could worsen host condition.

Interestingly, we found that the abundance of *Bifidobacterium* sp. was positively associated with survival, as previously discussed [17]. We found several other microbial species to be associated with survival that have been described to have anti-inflammatory properties. For instance, *Blautia faecis*, *C. aerofaciens* and *F. prausnitzii* are butyrate-producing bacteria that alleviate inflammatory disease and are associated with clinical remission in ulcerative colitis or Crohn’s disease patients [23–26]. The two latter ones (*C. aerofaciens* and *F. prausnitzii*) are also associated with clinical response to immunotherapy (anti-CTLA-4 or anti-PD-1 treatments) in cancer patients [27, 28]. *C. aerofaciens* is also associated with faecal microbiota transplantation efficacy to treat recurrent *Clostridioides difficile* infection [29], a bacteria known to cause potentially lethal colitis which occurs in frailer patients and abundance of which was associated with mortality in this study.

In addition to *Clostridioides difficile*, several microbial species known to have pro-inflammatory properties were positively associated with mortality. In fact, *Staphylococcus haemolyticus* abundance in gut microbiota is increased in patients with active coeliac disease compared to control subjects [30]. Many species belonging to *Ruminococcus genus* are associated with intestinal inflammation in ulcerative colitis [31]; *Campylobacter ureolyticus* is associated with acute and prolonged gastroenteritis and is implicated in the development of inflammatory bowel diseases [32]. *Malassezia* sp. is associated with cystic fibrosis lung exacerbation [33] and with intestinal inflammation in Crohn’s disease patients [34]. *Saccharomyces cerevisiae* is also associated with intestinal inflammation in celiac disease [35].

The main limitation of this study is the relative small number of patients and its monocentric design. The possible prognostic value of both gut bacteriobiota and mycobiota in critically ill patients should be addressed in large multicentre cohort studies to assess if it could be of interest alongside of SAPSII score which is much more convenient to perform than microbiome sequencing. Furthermore, our analysis assessed for linear but not for nonlinear association of variables with mortality outcome. Thanks to the development of machine learning, complex logistic regression model or different models tested in ensemble modelling could address non-linearity automatically without pre-specification [36]. Another limitation of our study is the lack of causality demonstration as stated for previous studies discussed above. In fact, gut microbiota composition is highly dependent on host condition—including age, sex, immune condition, frailty—[3, 37] which could be confounding factors underlying correlation but not causality. To get beyond the association links provided in this study, animal or organoïd models are needed to decipher the causality between gut microbiota and mortality through the modulation of the host condition. Confirmation of a causal link would enhance the hypothesis that gut microbiota modulation could be a therapeutic approach in ICU patients. If so, in vitro studies will be also required to identify the underlying mechanisms as concerns exist about the translocation of probiotics given to ICU patients who often have increased gut permeability [38].

## Conclusion

The gut bacteriobiota and mycobiota *α* diversities of critically ill patients are significantly decreased in non-survivors than in survivors and are independently associated with Day-28 mortality. The causal nature of this interference and, if so, the underlying mechanisms should be further investigated to assess if gut microbiota modulation could be a future therapeutic approach in critically ill patients.

## Supplementary Information

**Additional file 1. Supplemental Figure 1**. Non metric Bray-curtis analysis of β-diversity of the V3-V4 sequencing run. XXA, B, and C samples: gut bacteriobiota samples. GXX: lung bacteriobiota samples. BlancV3-V4: negative control. Mock: mock community.** Supplemental Figure 2**. Non metric Bray-curtis analysis of β-diversity of ITS2 sequencing run. XXA, B, and C samples: gut mycobiota samples. GXX: lung mycobiota samples. Blanc1 and blanc2: negative controls. Mock: mock community.

## Data Availability

The 16S rRNA gene and ITS2 sequences have been submitted to the European Nucleotide Archive (Accession N◦ ERP134948).

## Abbreviations

AKI: Acute kidney injury
ARDS: Acute respiratory distress syndrome
AUC: Area under the curve
ICU: Intensive care unit
IQR: Interquartile range
ITS: Internal transcribed spacer 2
LDA: Linear discriminant analysis
LefSe: Linear discriminant analysis size effect
NPV: Negative predictive value
OR: Odds ratio
PPV: Positive predictive value
ROC: Receiver operating characteristic
SAPSII: Simplified acute physiology score II
Se: Sensitivity
Sp: Specificity
16S rDNA: DNA region coding for ribosomal 16S RNA subunit

## Code availability

The scripts used for bioinformatics analysis during the current study are available from the corresponding author on reasonable request.
