# Supplementary material for: Gut bacteriobiota and mycobiota are both associated with Day-28 mortality among critically ill patients
Source: Crit Care. 2022 Apr 13;26:105. doi: 10.1186/s13054-022-03980-8 (PMC9007252; doi:10.1186/s13054-022-03980-8)

**Supplemental Figure 1** Non metric Bray-curtis analysis of β-diversity of the V3-V4 sequencing run. XXA, B, and C samples: gut bacteriobiota samples. GXX: lung bacteriobiota samples. BlancV3-V4: negative control. Mock: mock community.


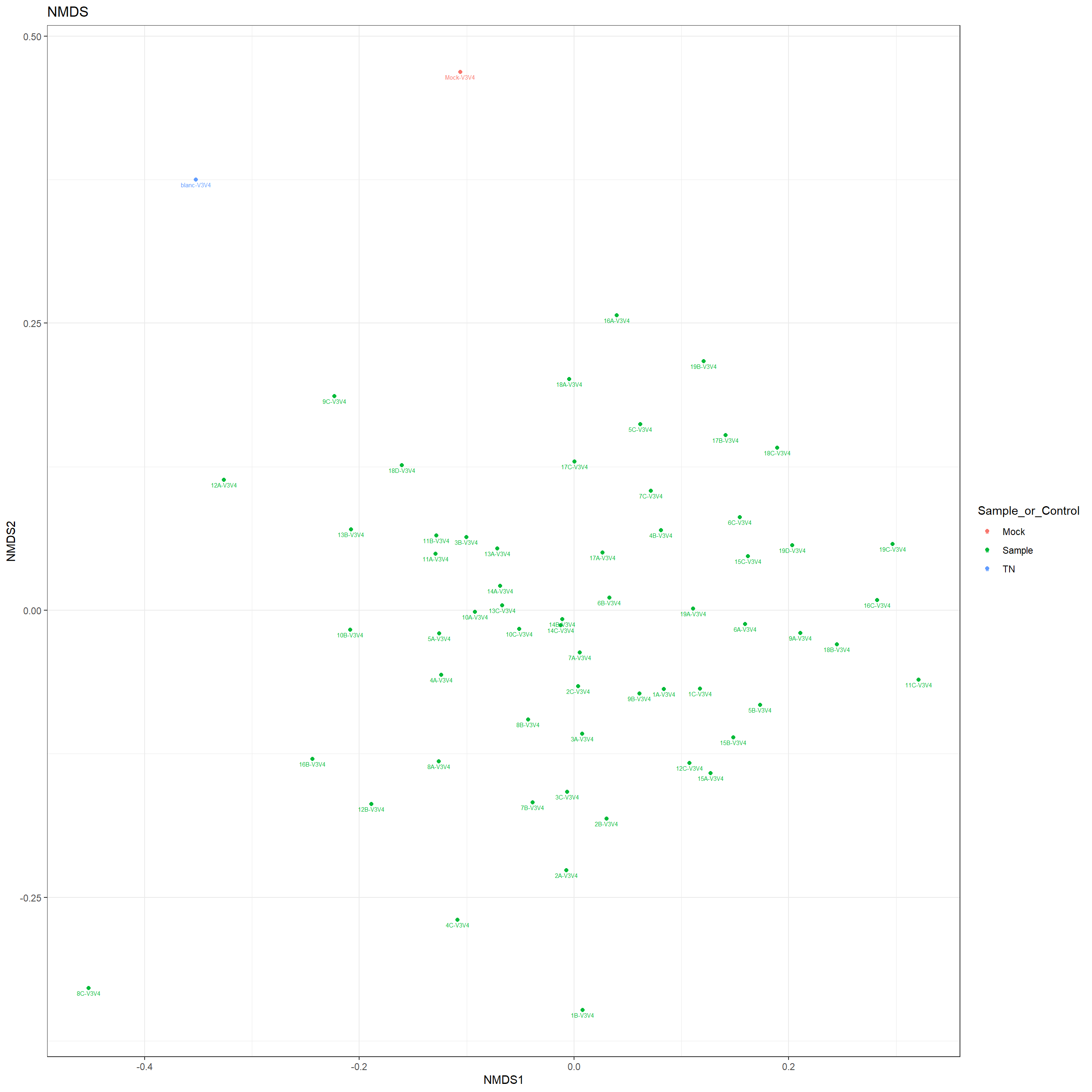


**Supplemental Figure 2** Non metric Bray-curtis analysis of β-diversity of ITS2 sequencing run. XXA, B, and C samples: gut mycobiota samples. GXX: lung mycobiota samples. Blanc1 and blanc2: negative controls. Mock: mock community.


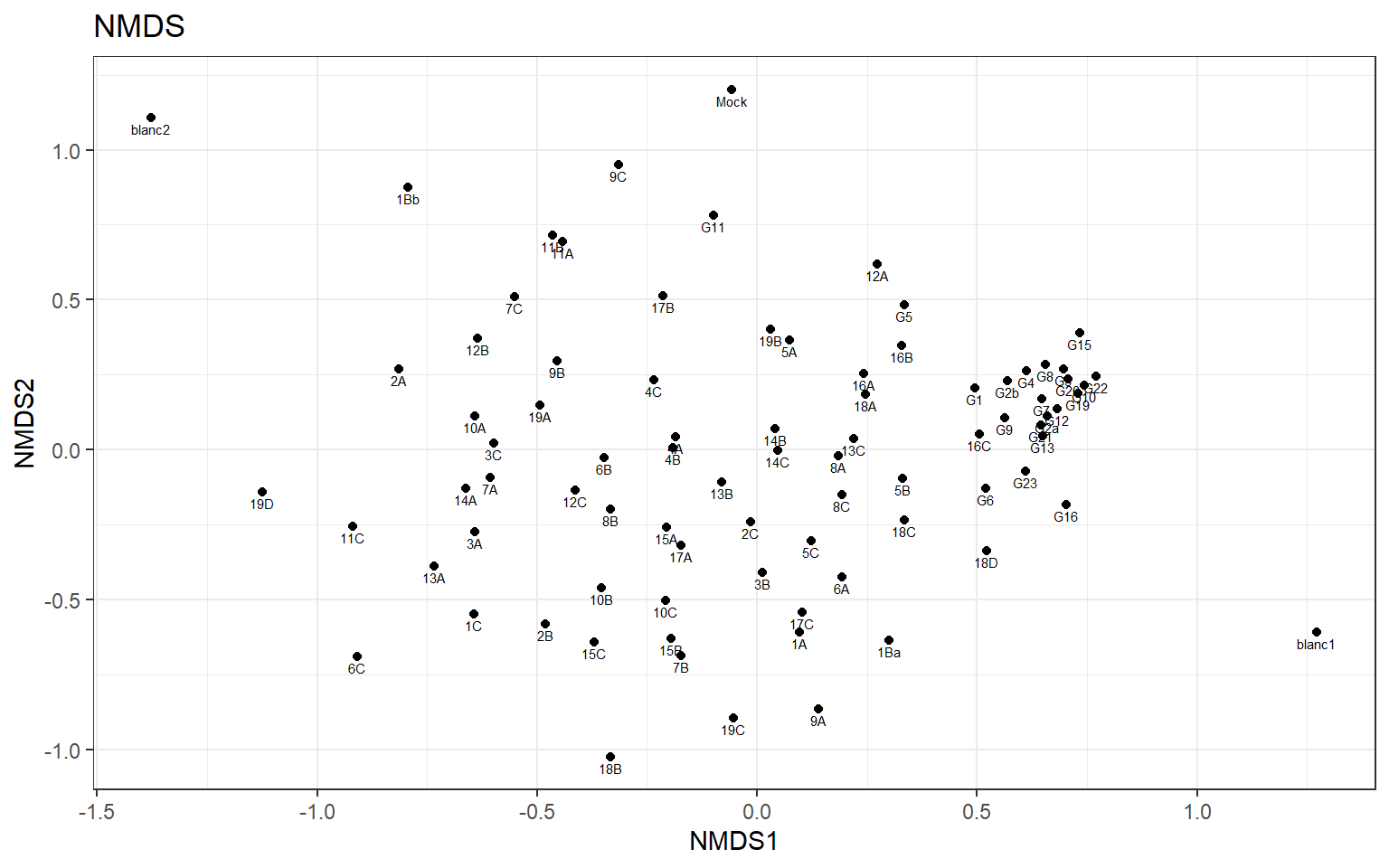

Supplement: Supplementary file 1 — Additional file 1. Supplemental Figure 1. Non metric Bray-curtis analysis of β-diversity of the V3-V4 sequencing run. XXA, B, and C samples: gut bacteriobiota samples. GXX: lung bacteriobiota samples. BlancV3-V4: negative control. Mock: mock community. Supplemental Figure 2. Non metric Bray-curtis analysis of β-diversity of ITS2 sequencing run. XXA, B, and C samples: gut mycobiota samples. GXX: lung mycobiota samples. Blanc1 and blanc2: negative controls. Mock: mock community. [file 13054_2022_3980_MOESM1_ESM.docx]
